# An Empirical Approach towards the Efficient and Optimal Production of Influenza-Neutralizing Ovine Polyclonal Antibodies Demonstrates That the Novel Adjuvant CoVaccine HT™ Is Functionally Superior to Freund's Adjuvant

**DOI:** 10.1371/journal.pone.0068895

**Published:** 2013-07-23

**Authors:** Natalie E. Stevens, Cara K. Fraser, Mohammed Alsharifi, Michael P. Brown, Kerrilyn R. Diener, John D. Hayball

**Affiliations:** 1 Experimental Therapeutics Laboratory, Hanson Institute, Adelaide, SA, Australia; 2 Sansom Institute, School of Pharmacy and Medical Science, University of South Australia, Adelaide, SA, Australia; 3 Preclinical, Imaging and Research Laboratories, South Australian Health and Medical Research Institute, Gilles Plains, SA, Australia; 4 School of Molecular and Biomedical Science, University of Adelaide, Adelaide, SA, Australia; 5 Cancer Clinical Trials Unit, Royal Adelaide Hospital, Adelaide, SA, Australia; 6 School of Medicine, University of Adelaide, Adelaide, SA, Australia; 7 School of Paediatrics and Reproductive Health, University of Adelaide, Adelaide, SA, Australia; University of Strathclyde, United Kingdom

## Abstract

Passive immunotherapies utilising polyclonal antibodies could have a valuable role in preventing and treating infectious diseases such as influenza, particularly in pandemic situations but also in immunocompromised populations such as the elderly, the chronically immunosuppressed, pregnant women, infants and those with chronic diseases. The aim of this study was to optimise current methods used to generate ovine polyclonal antibodies. Polyclonal antibodies to baculovirus-expressed recombinant influenza haemagglutinin from A/Puerto Rico/8/1934 H1N1 (PR8) were elicited in sheep using various immunisation regimens designed to investigate the priming immunisation route, adjuvant formulation, sheep age, and antigen dose, and to empirically ascertain which combination maximised antibody output. The novel adjuvant CoVaccine HT™ was compared to Freund’s adjuvant which is currently the adjuvant of choice for commercial production of ovine polyclonal Fab therapies. CoVaccine HT™ induced significantly higher titres of functional ovine anti-haemagglutinin IgG than Freund’s adjuvant but with fewer side effects, including reduced site reactions. Polyclonal hyperimmune sheep sera effectively neutralised influenza virus *in vitro* and, when given before or after influenza virus challenge, prevented the death of infected mice. Neither the age of the sheep nor the route of antigen administration appeared to influence antibody titre. Moreover, reducing the administrated dose of haemagglutinin antigen minimally affected antibody titre. Together, these results suggest a cost effective way of producing high and sustained yields of functional ovine polyclonal antibodies specifically for the prevention and treatment of globally significant diseases.

## Introduction

Antigen-specific polyclonal antibodies are generated for a wide array of purposes which range from fundamental laboratory studies and protocols to passive immunotherapy for life threatening conditions including snake envenomation [1] and drug toxicity [2]. Sheep are particularly attractive for the generation of passive polyclonal immunotherapeutics as ovine antibody fragments have demonstrated reduced immunogenicity and more consistent biological function than those derived from other animals [3]. Furthermore, large quantities of serum can be repeatedly obtained from sheep, with reduced maintenance costs and lower immune boosting demands than other large animals such as horses [4]. Specifically, polyclonal ovine antibodies in the form of antigen-binding antibody fragments or ‘Fab’ underlie the use of antibodies in critical care situations such as snake envenomation and digoxin toxicity [5]. Potential hypersensitivity reactions often associated with administration of whole antibody are considerably reduced by using these Fab fragments or their divalent counterpart F(ab)_2_. Hence, this type of treatment has the potential to be readily transferable to infectious disease management, particularly in light of the increased incidence of drug resistance to circulating pathogens [6], [7] and the medley of undesirable side effects often associated with conventional drug treatments [8], [9], [10]. Of particular interest here is the applicability of this approach to infections with viral pathogens such as influenza, as natural immunity to many such viruses is facilitated through the action of neutralising antibodies [3], [11], [12].

Whilst traditional vaccination reduces influenza-associated mortality [13], it is least efficacious in the immunocompromised individuals who are most susceptible to complications and increased mortality [14], [15], and who include pregnant women [16]. Consequently, immunocompromised individuals make up the majority of the many thousands of annual influenza-related deaths [14], [17], which provides the rationale for passive immunotherapy as influenza prophylaxis or treatment in these individuals because additional time is not needed to generate an efficient vaccine-induced adaptive immune response [18]. Indeed, passively administered influenza-specific antibody has been shown to inhibit influenza-induced mortality in rodents [19], although the selection of a suitable clinically applicable passive immunotherapeutic will be determined by its inherent neutralisation capacity, its safety as well as its commercial scalability and overall cost effectiveness [20]. These factors highlight the requirement for optimal efficiency at every stage of the production process.

Whilst downstream processing methods for existing commercial ovine polyclonal antibody preparations have been methodically optimised [21], [22], [23], there has been limited investigation into the best way to generate maximal antibody titres and overall yield of effective antibody from the sheep themselves. Indeed, there are few reports in the published literature directly comparing the parameters that can influence humoral immune responses in sheep [24]. This is particularly important considering that route of administration, antigen dose and adjuvant are well recognised as critical parameters in antibody production from other species [25], [26].

The route of immunisation can influence the induction of the humoral immune responses [27] by dictating which population of dendritic cells (DCs) interacts with antigen [28], [29]. For instance, subcutaneous immunisation is routinely applied commercially to produce hyperimmune ovine sera [22], [23] and facilitates antigen interaction with skin-associated DCs, including Langerhans cells, conventional DCs and macrophage-derived DCs [30]. Alternatively, intraperitoneal immunisation promotes antigen interaction with conventional DCs macrophages and plasmacytoid DCs, which may be beneficial depending on the antigen type [31], [32]. The functionality of site-specific DC subsets in sheep has not been well studied and thus empirical assessment is required to determine an optimal immunisation route. Antigen dose can also influence the outcome of immunisation; too little antigen may elicit inefficient responses [33], and too much antigen can promote adverse effects and immunotolerance [24]. The standard antigen dose used in generating ovine antisera varies widely (from micrograms to as much as five grams per animal [24]) and this uncertainty necessitates investigation of appropriate antigen dosage for optimal antibody output. The choice of adjuvant is another key factor in dictating both the quality and quantity of the humoral immune response [25], [34]. Adjuvants prolong and augment the effects of vaccination through various mechanisms which include increasing antigen persistence, stimulation of local inflammation, upregulation of cytokines and immunomodulatory factors, activation of phagocytic cells and promotion of antigen presentation [35], [36]. The current gold-standard for generation of a humoral immune responses in animals is Freund’s adjuvant (FA), a water-in-oil emulsion which is available in ‘complete’ or ‘incomplete’ formulations, that is, with or without heat-killed *Mycobacterium tuberculosis* respectively [37]. This adjuvant primarily works by increasing antigen persistence in the tissues [38] and stimulating a foreign-body reaction which results in local inflammation and recruitment of innate and adaptive immune cells [39]. It is however often associated with the formation of painful granulomas and can also result in tuberculin-type hypersensitivity [40]. It is not approved for human use and concerns with animal welfare have prompted investigation into alternative adjuvants for use in animals [39].

The current study applies empirical methods to optimise anti-A/Puerto Rico/8/1934 H1N1 (PR8) haemagglutinin (HA) ovine polyclonal antibody production using FA and a proprietary experimental adjuvant, CoVaccine HT™ (CV) [36], [41], [42], [43]. CoVaccine HT™ is in clinical development and consists of sucrose fatty acid sulphate esters (SFASE) immobilised on the oil droplets of a submicron emulsion of squalane-in-water [41]. We show that the age of sheep, the prime immunisation route, and dose has minimal impact on the antibody titre, although CV adjuvant induced superior anti-HA antibody titres than immunisation regimens incorporating FA. The influenza-neutralisation capacity of the anti-HA antibody was subsequently assessed by haemagglutination-inhibition (HAI) assays and was found to correlate closely with the antibody titres measured for each of the variables. Finally, it was demonstrated that high titre ovine hyperimmune serum can be successfully applied in a lethal *in vivo* murine influenza challenge model to prevent mortality in both a prophylactic and therapeutic context.

## Materials and Methods

### Recombinant Haemagglutinin Production

The HA sequence of A/Puerto Rico/8/1934 H1N1 (PR8) H1N1 (NCBI accession: AF389118) from PR8-transfected AB1 malignant melanoma cells (AB1-HA) was PCR cloned into pGEM-T Easy Vector system (Promega) using primers with appropriate restriction sites and a C-terminal 6x-His tag. Confirmed HA sequence was subsequently used to produce recombinant baculovirus using the Bac-to-Bac expression system (Invitrogen). Insect cells (Sf21) were maintained in sf900 SFM III (Invitrogen) supplemented with L-glutamine (100 µg/ml), penicillin (100 U/ml), gentamycin (100 µg/ml) and HEPES (10 mM, pH 7.2) in roller bottle flasks at 27°C. Protein was produced by infection of Sf21 insect cells 96 hours prior to protein harvest, and cultures were supplemented with 100 µg/ml L-glutamine 24 hours prior to harvest. Expressed protein was purified from cell lysate using Ni-NTA Agarose beads (Qiagen) according to the manufacturer’s instructions before dialysis with PBS. Protein purity was analysed by SDS-PAGE and anti-HA Western Blot and concentration determined with BCA assay.

### Immunisation and Sampling

Purified recombinant HA (rHA) antigen was diluted in PBS and emulsified with an equal volume of complete FA for prime immunisation or incomplete FA for boost immunisation (Gibco). Groups of five 9-month old or 3-year old Border Leicester x Merino ewes were immunised with 200 or 20 µg rHA in 4 ml emulsion, either subcutaneously (SC) at 4 axillary sites or as a bolus intraperitoneal (IP) injection (prime only). Alternatively, antigen in PBS was gently mixed with an equal quantity of CV suspension (Protherics Medicines Ltd) and sheep were immunised with 2 ml emulsion SC at 4 axillary sites. Sheep were administered a prime immunisation and a boost dose every 14 days for a total of five boosts. Serum was sampled prior to each immunisation and stored at −20°C.

### Anti-HA Antibody ELISA

Briefly, EIA/RIA high-binding ELISA plates (Costar) were coated with 10 µg/ml rHA overnight at 4°C. Plates were blocked with 2% (w/v) BSA (1 hour, 37°C) and pre-immune or hyperimmune sheep serum diluted as indicated in Figure S1 was added to duplicate wells and incubated for 2 hours (37°C). Bound ovine antibodies were detected with HRP-linked anti-ovine IgG antibody (Sigma; 1 hour, 37°C). The plates were developed with OPD substrate (Sigma), the reaction stopped with 3 M HCl and the absorbance read at 490 nm. Absorbance readings from negative control wells were subtracted from all readings. ELISA analysis of serially diluted samples indicated that a dilution of 1/50,000 could enable accurate comparison of a range of samples in the linear portion of the curve. A representative figure has been included in Figure S1.

### Haemagglutination-inhibition (HAI) Assay

Diluted pre-immune or hyperimmune serum samples (1/400) were pre-treated with chicken red blood cells (cRBC) to remove non-specific agglutinins (2 hours, room temperature) and the HAI assay was performed using a previously described method [44]. Briefly, treated serum (10 µl) was serially diluted 1/4 in duplicate wells of a round-bottom 96-well plate before the addition of PR8 influenza virus (5 haemagglutination units in 30 µl). After 30 minutes incubation at room temperature, 0.5% (v/v) cRBC in PBS (30 µl) was added to each well and gently mixed. Plates were visualised over a light box after 45 minutes. The endpoint HAI titre was recorded as the highest dilution of serum that was able to completely inhibit the agglutination of cRBC by virus in duplicate wells. Potential non-specific inhibition was discounted by the use of receptor-destroying enzyme (Table S1).

### Murine Model of Influenza Infection

Female 6–8 week old BALB/c mice were housed in PC2 defined pathogen-free conditions following institutional guidelines. Groups of 5 mice were administered high titre anti-HA ovine serum (1 ml) or PBS intraperitoneally, and challenged intranasally 24 hours later with 500 TCID_50_ PR8 influenza virus (32 µl). In the treatment models, mice were challenged with virus twenty-four hours prior to intraperitoneal administration of whole or diluted serum or control PBS as a 1 ml injection. Weight and clinical score of the mice were monitored and mice were euthanased upon achieving 20% (w/w) weight loss.

### Statistical Analysis

All statistical analysis was performed with GraphPad Prism V4.00 software.

### Ethics Statement

All animal experiments were approved by the SA Pathology/CHN and, where appropriate, Primary Industries and Resources South Australia animal ethics committees. All experiments were conducted in accordance with National and Institutional ethical guidelines.

## Results

### Intraperitoneal or Subcutaneous Prime Immunisation Induces Similar Peak Anti-Haemagglutinin Antibody Titres

Generally, antigen emulsified with FA is administered subcutaneously over multiple sites. However, it has been previously demonstrated that intraperitoneal prime immunisation can yield significantly higher antibody titres [45], [46]. Therefore, to directly compare these routes of prime immunisation, groups of sheep were immunised either subcutaneously (four sites) or intraperitoneally (bolus injection) with rHA emulsified in complete FA. Both groups were boosted subcutaneously every two weeks with rHA in incomplete FA for a total of five boosts.

**Figure 1:**
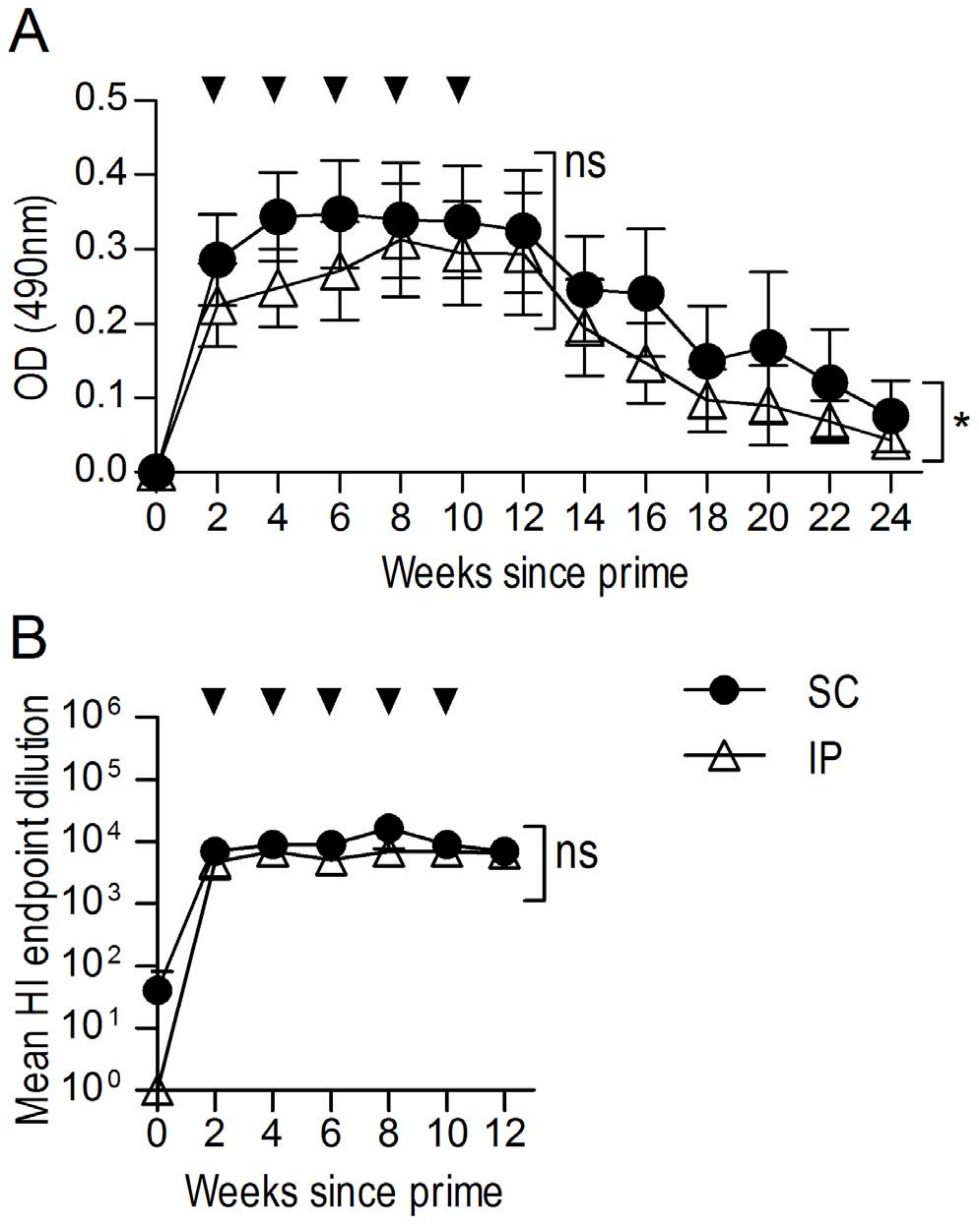
Different routes of prime immunisation yield similar anti-HA antibody titres. Sheep (n = 5) were immunised with 200 µg rHA SC or IP in complete FA. Sheep were boosted SC every two weeks to a total of five boosts in incomplete FA (indicated by arrows). Pre-immune (time 0) or hyperimmune serum samples were analysed for anti-HA IgG via ELISA (1/50, 000 dilution) (A), and HAI(B). Data are expressed as the mean ± SEM. Two-way repeated-measures ANOVA was applied to evaluate significance which is denoted as thus: * = P<0.05, ** = P<0.01, *** = P<0.001, ns = not significant.

Serum samples were collected fortnightly and the titre of anti-HA immunoglobulin G (IgG) was analysed by ELISA. Assessment of results by two-way repeated-measures ANOVA indicated no significant difference between antibody titres during the induction phase (weeks 2–12) of the immune response (Figure 1A). However when ELISA data from all time points including twelve weeks post-immunisation were analysed, a significant trend for higher antibody titres was observed in the subcutaneously primed group (Figure 1A; P<0.05), which may indicate a slower decline in antibody titre for this group. Importantly, neither group exhibited a significant difference in the ability of various serum dilutions to inhibit haemagglutination of PR8 in an HAI assay (Figure 1B). Based on these results, the subcutaneous prime immunisation was selected for all subsequent comparisons.

### CoVaccine HT™ Elicits Significantly Higher Ovine Anti-haemagglutinin Antibody Titres than Freund’s Adjuvant

The experimental adjuvant CoVaccine HT™ is an oil-in-water emulsion, which is designed with synthetic carbohydrate structures on squalane microdroplets and which produces proinflammatory responses through interaction with innate immune receptors, including TLR-4 [41], [47]. This adjuvant offers the advantage of presenting amphipathic membrane target antigens in native formation due to the squalane-in-water formulation. It has shown efficacy in combination with a range of antigens including malarial antigens [48], influenza glycoproteins [47] and gonadatropin-releasing hormone [49]. In order to determine if CV is capable of inducing anti-HA antibody responses comparable to those generated with FA, sheep were primed with rHA either traditionally emulsified with complete FA or gently mixed with CV and boosted fortnightly with rHA in incomplete FA or CV respectively. Serum samples were collected fortnightly and analysed via ELISA and HAI assay.

**Figure 2:**
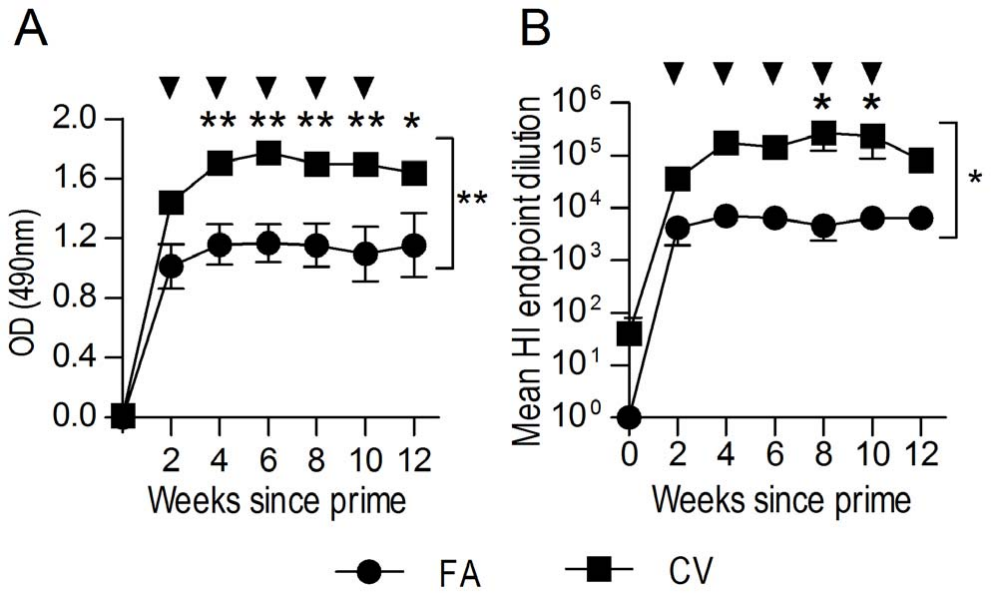
CoVaccine HT™ adjuvant elicits significantly higher anti-HA antibody titres than Freund’s adjuvant. Sheep (n = 5) were immunised SC with rHA (200 µg) in complete FA or CoVaccine HT™ (CV). Sheep were boosted similarly every two weeks (five boosts indicated by arrows) with rHA in incomplete FA or CV. Pre-immune (time 0) or hyperimmune serum samples were analysed for anti-rHA IgG via ELISA (1/50, 000 dilution) (A) and HAI (B). Data are represented as the mean ± SEM. Two-way repeated-measures ANOVA with Bonferroni post-test was applied to evaluate significance which is denoted as thus: * = P<0.05, ** = P<0.01, *** = P<0.001, ns = not significant.

Two-way repeated-measures ANOVA and post-test analysis indicated that CV elicited significantly higher antibody titres overall (P<0.01) and at multiple time-points post prime (P<0.05) compared to FA (Figure 2A). Consistent with the ELISA results, the CV group sera demonstrated significantly higher HAI capacity than those from the FA group (P<0.05; Figure 2B), suggesting that CV may be a good alternative to FA in future immunisation regimens.

### Age of Sheep did not Significantly Alter the Ability to Generate High Anti- haemagglutinin Antibody Titres

**Figure 3:**
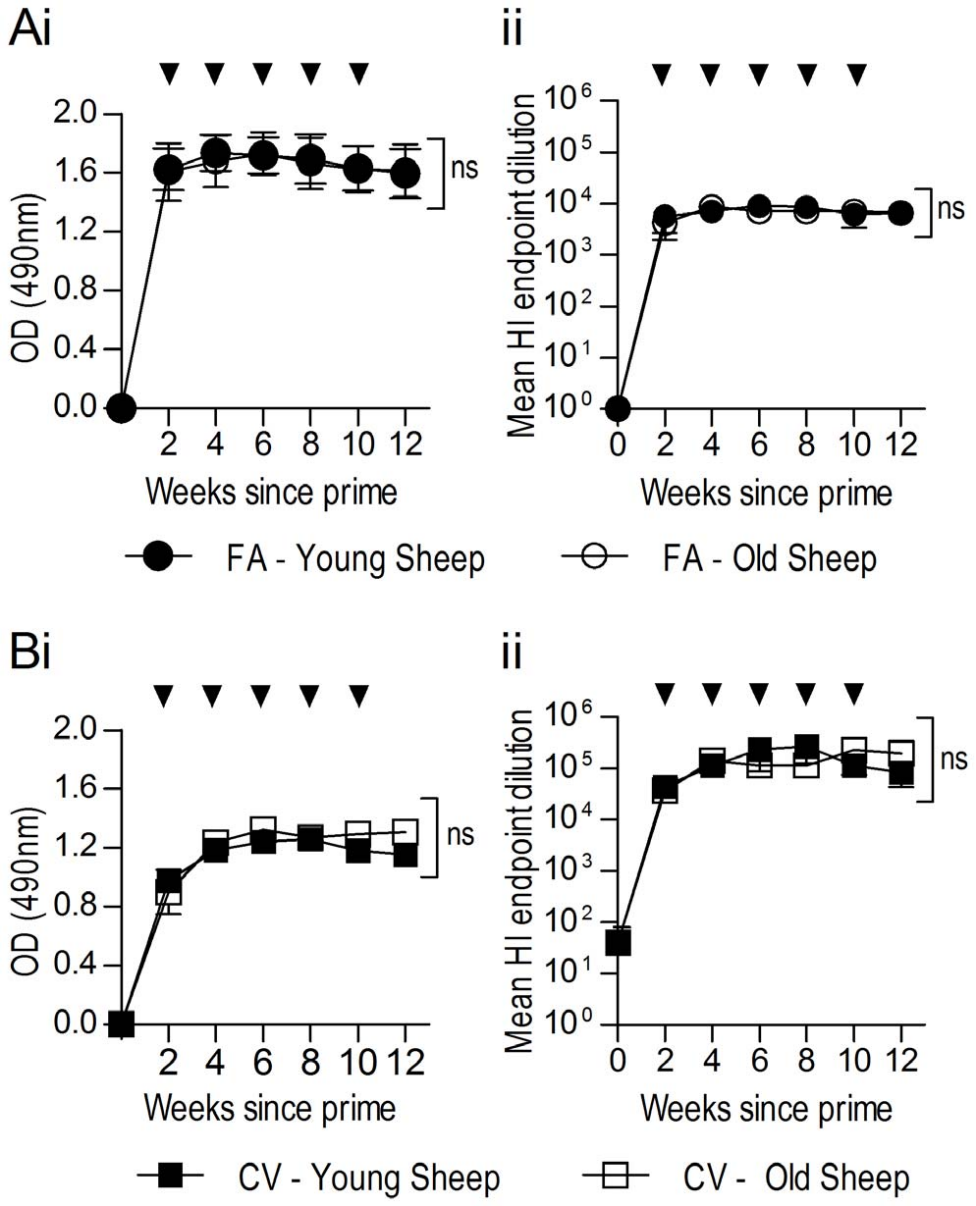
Nine-month old and three-year old sheep produce similar anti-HA antibody titres. Sheep (n = 5) at either nine months (young) or three years (old) were immunised SC with 200 µg of rHA in complete FA (A) or CV (B). Sheep were subsequently boosted SC every two weeks to a total of five boosts in incomplete FA or CV (indicated by arrows). Pre-immune (time 0) or hyperimmune serum samples were analysed for anti-rHA IgG via ELISA (1/50, 000 dilution) (Ai, Bi) and HAI (Aii, Bii). Data are expressed as the mean ± SEM. Two-way repeated-measures ANOVA with Bonferroni post-test was applied to evaluate significance; ns = not significant.

It was hypothesised that younger sheep may produce higher antibody titres than older sheep due to a potential age-related decline in immunity in aged sheep. To assess the effect of sheep age on antibody output, sheep at nine months or three years of age were immunised subcutaneously with rHA either in complete/incomplete FA or CV as described above. Serum samples taken every two weeks were assessed by ELISA and HAI assays (Figure 3). Analysis of ELISA results by two-way repeated-measures ANOVA with Bonferroni post-tests revealed no significant difference between antibody titres overall, or at individual time points for serum produced in young or old sheep by administration of rHA emulsified in FA (Figure 3Ai) or CV (Figure 3Bi). Similarly, there was no significant difference in the neutralising activity of anti-HA serum from different aged sheep in HAI assays in either the FA-immunised (Figure 3Aii) or CV-immunised groups (Figure 3Bii). Consistent with previous results, HAI titres for either old or young sheep immunised with rHA-FA emulsion were significantly lower (P = 0.001) than the corresponding CV group (Figure 3Aii and Figure 3Bii respectively). These results suggest that age does not impact on the quality of the antigen-induced antibody response.

### A Ten-fold Lower Dosage of Antigen did not Yield Significantly Lower Anti-haemagglutinin Antibody Titres

**Figure 4:**
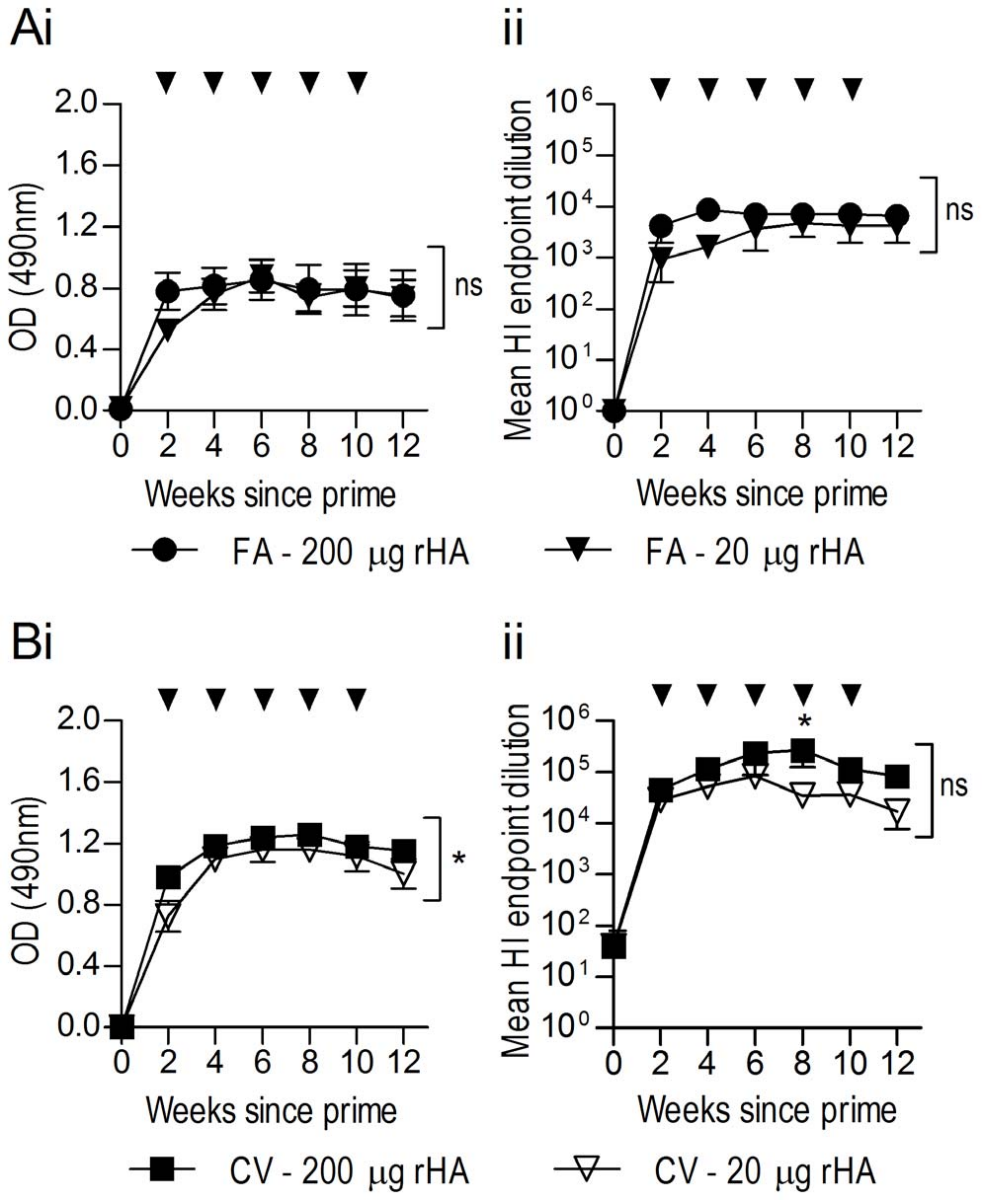
Low antigen dose produces similar anti-HA antibody titres. Sheep (n = 5) were immunised SC with 200 or 20 µg of rHA in complete FA (A) or CV (B). Sheep were then boosted SC every two weeks to a total of five boosts in incomplete FA or CV (indicated by arrows). Pre-immune (time 0) or hyperimmune serum samples at a 1/50,000 dilution were analysed for anti-HA IgG via ELISA (Ai, Bi) and HAI (Aii, Bii). Data are represented as the mean ± SEM endpoint dilution. Data of both assays were analysed by two-way repeated-measures ANOVA with Bonferroni post-tests; significance is denoted as thus: * = P<0.05, ** = P<0.01, *** = P<0.001, ns = not significant.

Cost of antigen is an important factor in any immunisation regimen, therefore to determine whether economic gains may be made by immunisation with a lower dose of antigen, sheep were immunised subcutaneously with 200 µg or 20 µg rHA either in FA or CV as previously described. Serum samples taken every two weeks were subsequently analysed by ELISA and HAI assays (Figure 4). Statistical scrutiny revealed no significant difference in antibody titre between high-dose and low-dose FA groups as indicated by ELISA (Figure 4Ai) and HAI results (Figure 4Aii). In comparison, significant differences were observed within the CV groups in both the ELISA assay (Figure 4Bi; P<0.05) and HAI assay (Figure 4Bii; P<0.05), however the magnitude of the decrease in the low-dose group was comparatively small. Indeed, analysis of individual time points by Bonferroni post-test comparisons revealed a significant difference in HAI titre at only one time point. Importantly, HAI results of CV group sera again revealed a more than ten-fold increase in mean endpoint serum dilution for both the high and low dose rHA over that observed with FA-induced sera (Figure 4Bii vs Figure 4Aii). These data supports the use of a lower antigen dose to elicit comparably high quantities of anti-HA antibody whilst reducing costs associated with antigen production.

### CoVaccine HT™ Induced Fewer Adverse Site Reactions as Compared to Freund’s Adjuvant

**Figure 5:**
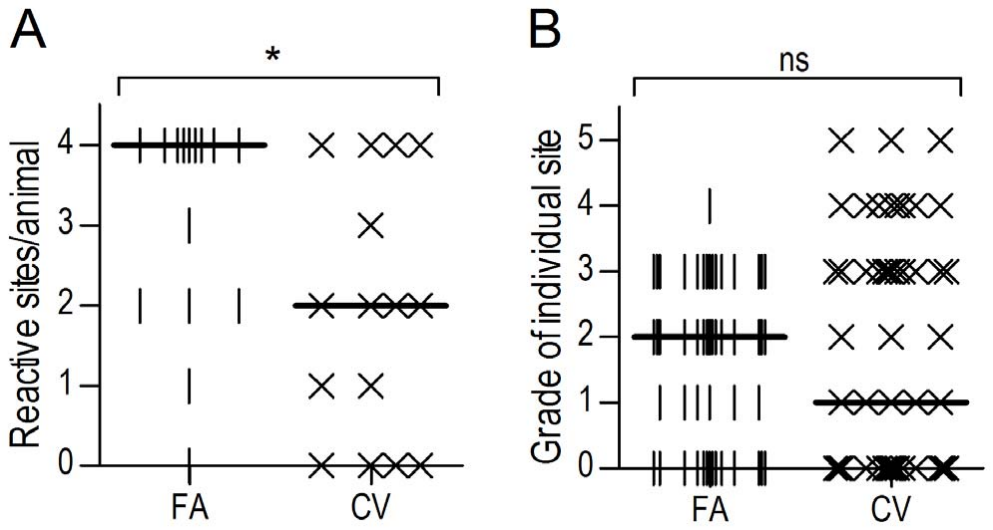
Repeated subcutaneous immunisation with CoVaccine HT™ elicits fewer reactive immunisation sites than Freund’s adjuvant. Sheep (n = 15) were immunised SC with rHA antigen either in complete/incomplete FA or CV. Two weeks following the final boost immunisation, injection sites were examined and palpable lumps were enumerated (A) and graded (B). A scoring system was devised based on the size and characteristics of the reaction sites to rank the level of reactivity of each individual site. Grades of site reactivity: 0– no reaction; 1– slight skin irregularity; 2– lump<10 mm diameter or larger skin irregularity; 3– multiple small lumps/single lump<40 mm; 4– lump<80 mm; 5–≥80 mm lump. The data was analysed by Mann-Whitney rank test; significance is denoted as thus: * = P<0.05, ** = P<0.01, *** = P<0.001, ns = not significant.

As FA immunisation often induces site-specific reactions, it was necessary to determine whether CV similarly induced local inflammatory reactions at the immunisation site. Observational data were collected twelve weeks post-prime where the number and size of palpable subcutaneous lumps at each site was recorded, and sites were given a rating on a graded system (denoted in Figure 5). No significant differences in size or number of reactive sites were observed within adjuvant groups in the age and antigen dose experiments (data not shown); therefore observational data from all sheep receiving each subcutaneous immunisation regimen were pooled and compared. Statistical analysis by Mann-Whitney rank test revealed a significant difference between the number of reactive sites, with sheep receiving rHA in FA exhibiting significantly more reactive sites per animal, many at all injection sites, than those sheep receiving antigen mixed with CV (Figure 5A; P<0.05). Interestingly, there was a trend toward lower reactivity scores of CV sites than of FA sites (Figure 5B), although altogether, the variation of CV reactivity scores was greater than (SD = 1.750) than that of FA scores (SD = 1.136).

### Prophylactic and Therapeutic Administration of Anti-haemagglutinin Hyperimmune Serum Protected Mice from a Lethal Influenza Challenge

**Figure 6:**
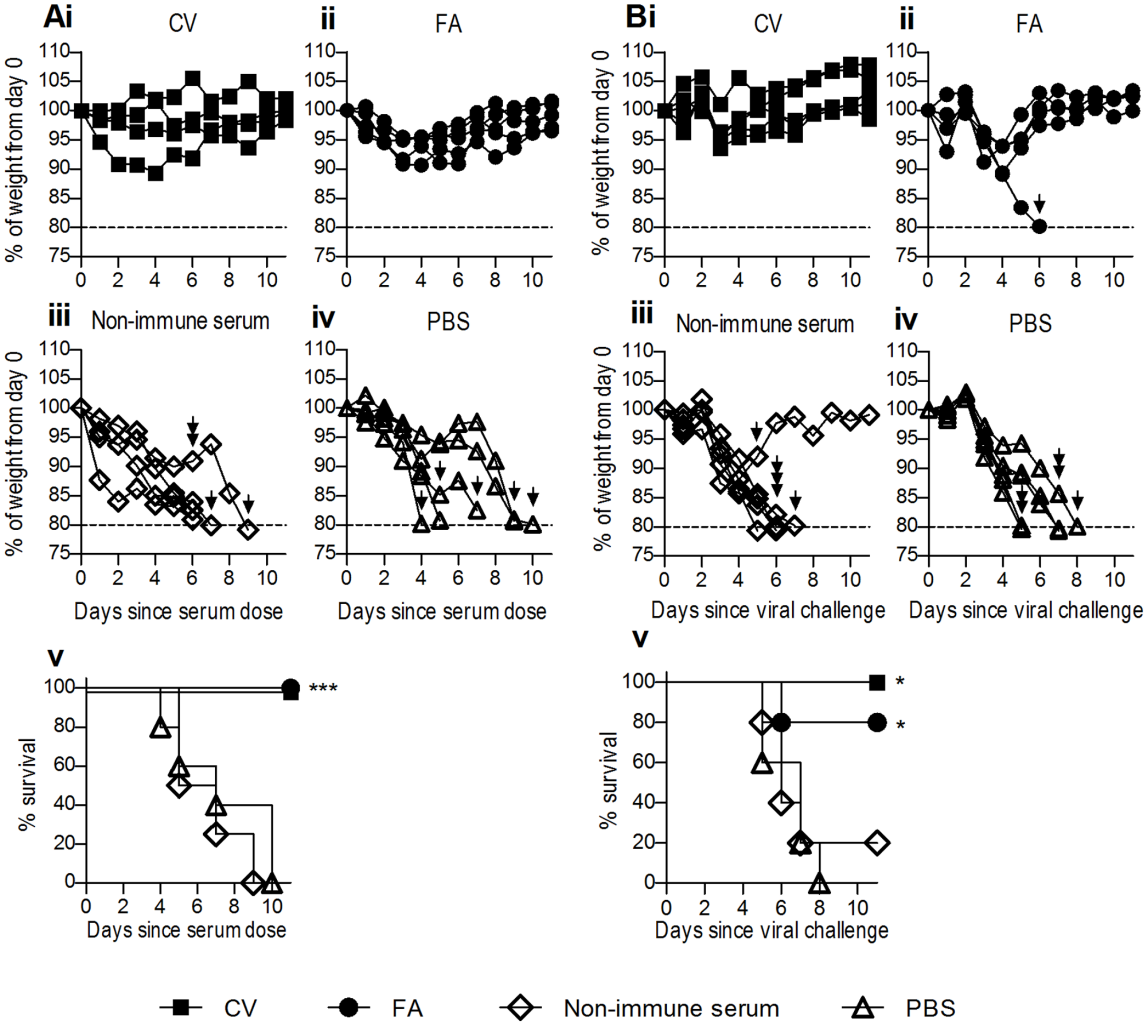
Prophylactic or therapeutic administration of ovine anti-HA serum is protective against lethal influenza challenge. Mice (n = 5) were prophylactically administered pooled serum (1 ml, IP) from either young sheep receiving 200 µg rHA SC in CV (Ai) or FA (Aii), day zero pre-bleeds from corresponding sheep from both groups (Aiii) or PBS as a control (Aiv). Twenty-four hours later mice were challenged with a lethal dose of PR8 (500 TCID_50_). Mice reaching a predetermined endpoint of 20% weight loss (dotted line) were euthanased as indicated by arrows. Mice (n = 5) were challenged with 500 TCID_50_ PR8 and twenty-four hours later therapeutically administered serum or PBS control as above (Bi–iv). In each panel, data show percentage weight loss of individual mice. Survival curves of mice are also shown (Av, Bv). Mantel-Cox survival analysis was performed on survival curves; significance between all curves is denoted as thus: * = P<0.05, ** = P<0.01, *** = P<0.001, ns = not significant.

Even though hyperimmune serum consistently prevented influenza-induced haemagglutination of RBC *in vitro*, we wished to determine the *in vivo* potential of this serum to prevent lethal influenza infection. To assess the protective ability of polyclonal anti-HA antibodies, mice received pooled hyperimmune serum from sheep immunised with rHA emulsified in CV or FA, or pooled non-immune serum or PBS diluent as controls. Twenty-four hours later, mice were challenged with 500 50% tissue-culture infective doses (TCID_50_) of live PR8 by intranasal administration and the clinical course was closely monitored (Figure 6A). Results indicated that mice which received prophylactic hyperimmune serum from either CV- or FA-immunised sheep exhibited some weight loss immediately after influenza infection, although this loss was quickly regained (Figure 6Ai and 6Aii respectively). In contrast, those mice that received non-immune serum or PBS had sustained weight loss and all had reached their pre-determined clinical endpoints by day 10 (Figure 6Aiii and 6Aiv respectively). Mantel-Cox survival analysis showed that both CV and FA hyperimmune serum prevented the lethal consequences of influenza infection in contrast to the effects of administration of non-immune serum or PBS (Figure 6Av; P<0.001).

To determine whether these polyclonal antibodies could treat an active influenza infection, hyperimmune or non-immune serum or PBS was administered twenty-four hours after viral challenge with 500 TCID_50_ PR8 (Figure 6B). Similarly, results indicated that infected mice which received hyperimmune serum therapy exhibited some weight loss before recovery (Figure 6Bi, 6Bii), whereas most mice that received non-immune serum or PBS had sustained weight loss and were euthanased by day 10 (Figure 6Biii, 6Biv). Similar to the effects of prophylactic administration of hyperimmune serum, Mantel-Cox survival analysis revealed a significant improvement in survival of the mice that received hyperimmune serum compared to control mice (Figure 6Bv; P<0.05). Taken together, it is clear that ovine hyperimmune serum can protect against and treat lethal influenza infections in mice.

### Hyperimmune Anti-haemagglutinin Serum Elicited with CoVaccine HT™ Exhibits Greater Potency in vivo as Compared to that Elicited by Freund’s Adjuvant

Anti-HA serum elicited with CV as an adjuvant had significantly better potency *in vitro* (Figure 2); to determine if this translated to increased potency *in vivo*, mice (n = 6) were intranasally inoculated with 500 TCID_50_ PR8 influenza and twenty-four hours later treated with different doses of high titre pooled serum (IP, equivalent to 1000, 500, 250 or 50 µl serum in a 1 ml injection) elicited with either CV or FA. Groups of mice received PBS or non-immune sheep serum as controls. Clinical disease course was monitored and euthanasia performed according to endpoints described above.

**Figure 7:**
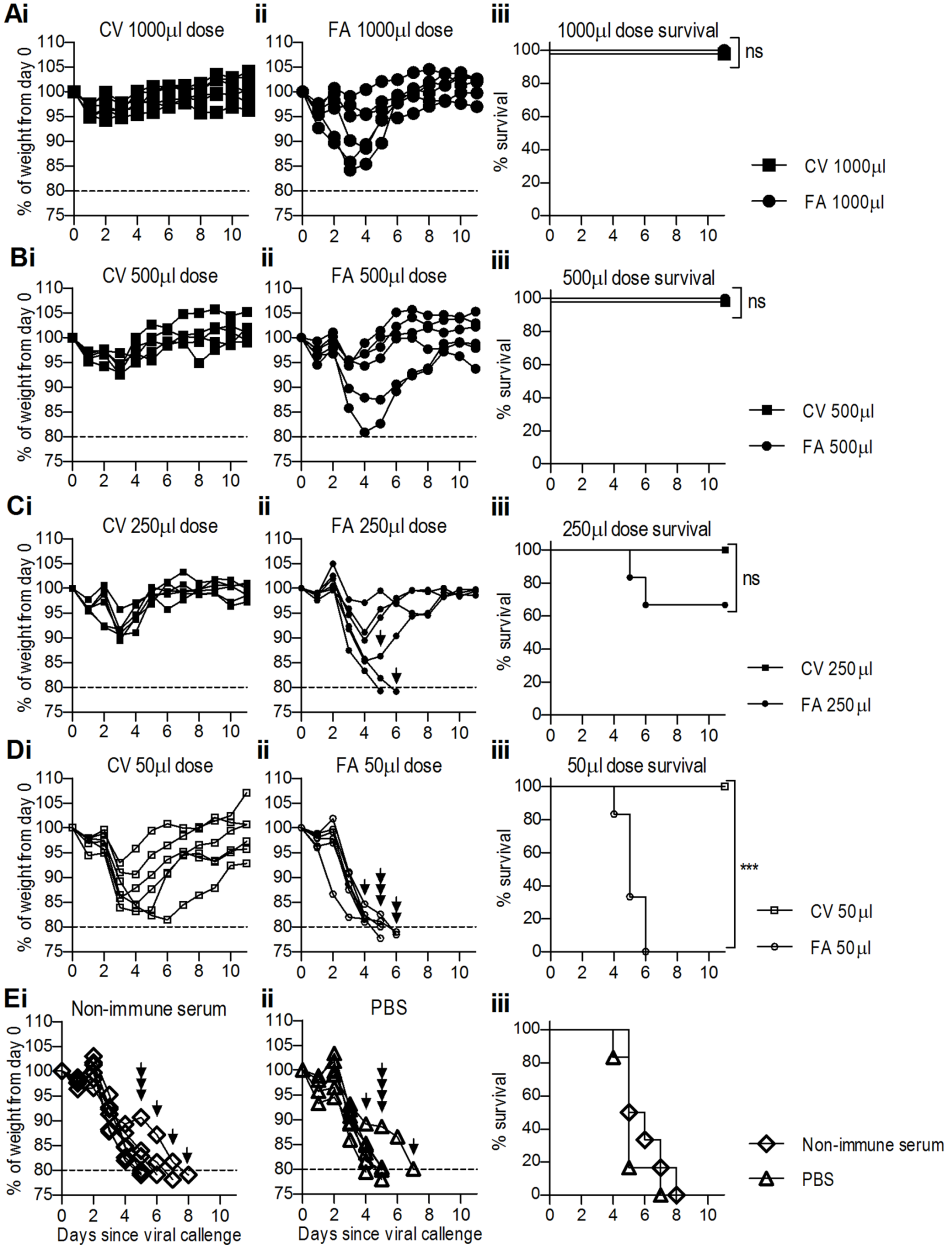
Ovine anti-HA serum elicited with CoVaccine HT™ adjuvant show greater *in vivo* potency as compared to that by Freund’s adjuvant. Mice (n = 6) were challenged with 500 TCID_50_ PR8 and twenty-four hours later therapeutically administered dose equivalents of 1000 (A), 500 (B), 250 (C) or 50 µl (D) of pooled hyperimmune serum (1 ml, IP) from sheep that received rHA antigen in CV (i) or FA (ii). Control groups of mice (n = 6) received non-immune serum (Ei) or PBS (Eii) twenty-four hours following viral challenge. Mice reaching a predetermined endpoint of 20% weight loss (dotted line) were euthanased as indicated by arrows. In each panel, data show percentage weight loss of individual mice. Survival curves of mice are also shown (iii). Mantel-Cox survival analysis was performed on survival curves; significance between survival curves is denoted as thus: * = P<0.05, ** = P<0.01, *** = P<0.001, ns = not significant.

Most notably, mice that had therapeutically received CV serum were completely protected from lethality from infection (Figure 7Ai–Di), regardless of dose, whereas the lower doses of 250 µl to 50 µl of FA serum were not completely protective (Figure 7Cii–Dii). Furthermore, although the 500 and 1000 µl FA serum dose protected mice from lethality of infection (Figure 7Aii–Bii) weight loss in these mice was more severe than that observed in mice receiving equivalent doses of CV serum (Figure 7Ai–Bi).

Mantel-Cox survival analysis revealed a significant difference between survival curves of all of the CV serum mice and controls (data not shown). Comparison of the survival curves of FA and CV serum mice revealed a significant survival advantage of CV serum at a 50 µl dose (Figure 7Diii; P<0.001). A minimum dose of 500 µl of FA serum was required for complete protection whereas a ten-fold lower dose of CV serum also offered complete protection. These data are consistent with the HI results that demonstrate an approximate ten-fold higher neutralising antibody titre in serum from sheep immunised with antigen in CV as compared to FA.

Taken together, these data suggest that lower doses of CV-elicited sera provide protection against influenza and that implementation of this adjuvant in ovine polyclonal antibody production could dramatically increase the efficiency of eliciting functional neutralising antibodies in sheep.

## Discussion

Ovine polyclonal antibodies are frequently generated for commercial and small laboratory scale applications, yet there has been limited investigation into maximising the amount of antibody generated through optimised ovine immunisation regimens. Within this study, methods to improve ovine antibody production were empirically investigated and demonstrated that such ovine polyclonal antibodies effectively bound influenza virus *in vitro* and prevented the death of mice infected with influenza virus.

The route of immunisation has been shown to influence the nature and intensity of the immune response generated to an antigen [29], [45], [46]. In the current study, subcutaneous prime immunisation with antigen in FA yielded significantly higher overall anti-HA antibody titres when compared to intraperitoneal prime. These findings are in contrast to an earlier study which demonstrated that anti-*C. parvum* antibody titres at parturition [45] were sixteen-fold higher in the serum when sheep received intraperitoneal immunisation compared to intramuscular immunisation. Furthermore, at twenty days post-lambing, detectable anti-*C*. *parvum* IgG had stabilised to approximately two-fold higher in the intraperitoneal-administered group. This discrepancy may be a consequence of altered immunological function in the sheep during pregnancy, or the differing nature of the antigens used [46]. It is possible that the rHA antigen is more efficiently presented by cells of the subcutaneous tissues than those in the peritoneal environment [28], [29], as CD14-positive DCs present in the dermis are directly involved in the differentiation of antibody-producing plasma cells [50], [51]. Furthermore, the hydrophobic properties of FA may enable the stable deposition of the antigen-adjuvant mixture at immunisation sites which is likely to be more favourable for long term immunity [52]. That is, the sheep subcutaneously primed may have had more local and stabilised antigen depots than the intraperitoneally-primed sheep as comparatively the peritoneal environment allows substances to disperse more freely.

The use of a novel experimental adjuvant CV was compared to FA, the adjuvant routinely used for the commercial and experimental production of polyclonal ovine antibodies. Interestingly, CV was found to raise significantly higher specific serum antibody titres than FA. The CV serum was also confirmed to exhibit higher potency *in vivo.* The enhanced efficacy of CV to induce high functional antibody titres may be partially attributable to the integrity of the antigen once mixed with this particular adjuvant. Protein antigens may be fully or partially denatured in water-in-oil adjuvants such as FA, which may lead to the deformation of native epitopes [53] resulting in antibodies with a poor affinity for the native protein. In contrast, CV is an oil-in-water emulsion of hydrophobic and negatively-charged sucrose fatty acid sulphate esters formulated with submicron emulsions of squalane that is combined with aqueous antigen and thus would result in less protein denaturation than FA [41]. The accessibility of the antigen to immune cells may also have contributed to increased efficacy of CV compared to FA. While the tendency of FA to form viscous deposits of antigen-adjuvant mixture in the tissues results in sustained slow-release of antigen, it may also result in limited antigen exposure to the cellular environment. In contrast, antigen mixed with CV is in an aqueous environment and this may facilitate dispersal into the tissues and increase the amount of antigen immediately accessible to antigen processing immune cells, thus increasing antibody titres. Indeed, recent preclinical studies have also shown that this adjuvant can enhance vaccine responses in not only sheep as described here but also in pigs [41], mice [42], [47], ferrets [36] and macaques [43]. The likely enhanced dispersal of CV as compared to FA may also explain why CV induced significantly fewer reactive sites that FA, which importantly suggests a superior safety profile to FA. These observations highlight CV as a safer and highly effective potential alternative to FA for adjuvanting antigens, particularly when factoring in the time-consuming and often hazardous emulsification periods needed with FA and other water-in-oil adjuvants prior to administration. Furthermore, these advantages combined with the ability to elicit a high titre IgG antibody response in a broad range of other animal species, favours the future practical application of CV to the rapid and large scale production of passive immunotherapeutics for veterinary and clinical use.

There were no significant age-related differences in the anti-HA antibody titres achieved after immunisation with either CV- or FA-rHA. Depending on size and growth characteristics, the quantity of blood routinely obtained from younger sheep may be upwards of a third less than that obtained from adult sheep [54]. However, the ability to utilise younger sheep for commercial polyclonal antibody production means that significant increases in antibody output over the lifespan of the animal would be possible, thus offsetting agistment costs much sooner. This would be particularly important if younger sheep were employed together with adjuvants such as CV that increase overall antibody titre. The costs associated with ovine antibody production could be minimised further if high antibody titres could be maintained with reduced amounts of costly antigen. Here, we showed that a ten-fold lower dose of antigen than that used conventionally resulted in only slightly lower (albeit significantly lower with CV adjuvant) anti-HA antibody titres in sheep. However, the magnitude of this difference is unlikely to matter particularly when compared to the difference in the amount of antigen used and instead reflects the complex relationship between antigen dosage and antibody titre [33]. Furthermore, the optimal antigen dosage will be affected by different adjuvants and antigens with correspondingly different pathways used for antigen processing, presentation and antibody responses [55]. Our results clearly indicate that the lower HA antigen dose induced anti-HA ovine antibody titres similar to the high titres induced with the higher antigen dose. Moreover, we expect that this reduced need for antigen will have the added benefit of markedly reducing production costs. Importantly, all immunisation regimens tested here resulted in the production of potent anti-HA antibodies that were able to treat and prevent lethal influenza infection in mice.

Antigenic variability among influenza strains remains one of the major challenges facing the development of an influenza-specific passive immunotherapeutic. However, it has been already been shown that it is possible to elicit strain cross-reactive anti-influenza hyperimmune serum by administering different subtypes of influenza haemagglutinin [56], [57]. In addition, administration of an antigen (whether it be a whole pathogen or parts thereof) to a live animal benefits from ‘natural selection’ by the host for the most immunogenic targets and mimics the natural immune response to infection or vaccination [58]. The widespread application of passive immunotherapy to treat influenza may be of particular benefit during pandemic outbreaks of the infection, such as the recent 2009 H1N1 pandemic [59]. During an influenza pandemic, a premium is attached to the timely and large-scale production and distribution of an effective therapy. Knowing best how to rapidly make at least cost large quantities of potent ovine neutralising antibodies against influenza virus or other potential pandemic pathogens could be an important contribution to reducing the disease burden and its associated societal and economic costs.

## Supporting Information

Figure S1 **Preliminary ELISA of anti-HA ovine serum samples.** An ELISA was performed on selected samples for assay development. Serial dilutions of pre-immune or hyperimmune sheep serum were added to duplicate wells of a rHA-coated ELISA plate and specific antibody was detected with HRP-linked anti-ovine IgG antibody. Signal was developed with OPD substrate until colour was visible in serum-free wells. Absorbance readings from blank wells were subtracted from all readings. The results indicated that a 1/50,000 serum dilution gave OD readings within the linear portion of the generated curve for hyperimmune samples. Consequently a 1/50,000 dilution was used in subsequent assays to assess experimental samples.
(TIF)

Table S1 **Assessment of the effects of Receptor Destroying Enzyme on Haemagglutination-inhibition endpoint titres.** In order to determine the effects of receptor-destroying enzyme (RDE) on endpoint HAI titres; selected serum samples were assayed with or without treatment with RDE (Sigma, 37°C, O/N). Samples were then treated with chicken red blood cells and assayed as described. Endpoint HAI titres were identical for all samples tested.
(DOCX)
