# Supplementary figures and images for: An Empirical Approach towards the Efficient and Optimal Production of Influenza-Neutralizing Ovine Polyclonal Antibodies Demonstrates That the Novel Adjuvant CoVaccine HT™ Is Functionally Superior to Freund's Adjuvant
Source: PLoS One. 2013 Jul 23;8(7):e68895. doi: 10.1371/journal.pone.0068895 (PMC3720891; doi:10.1371/journal.pone.0068895)

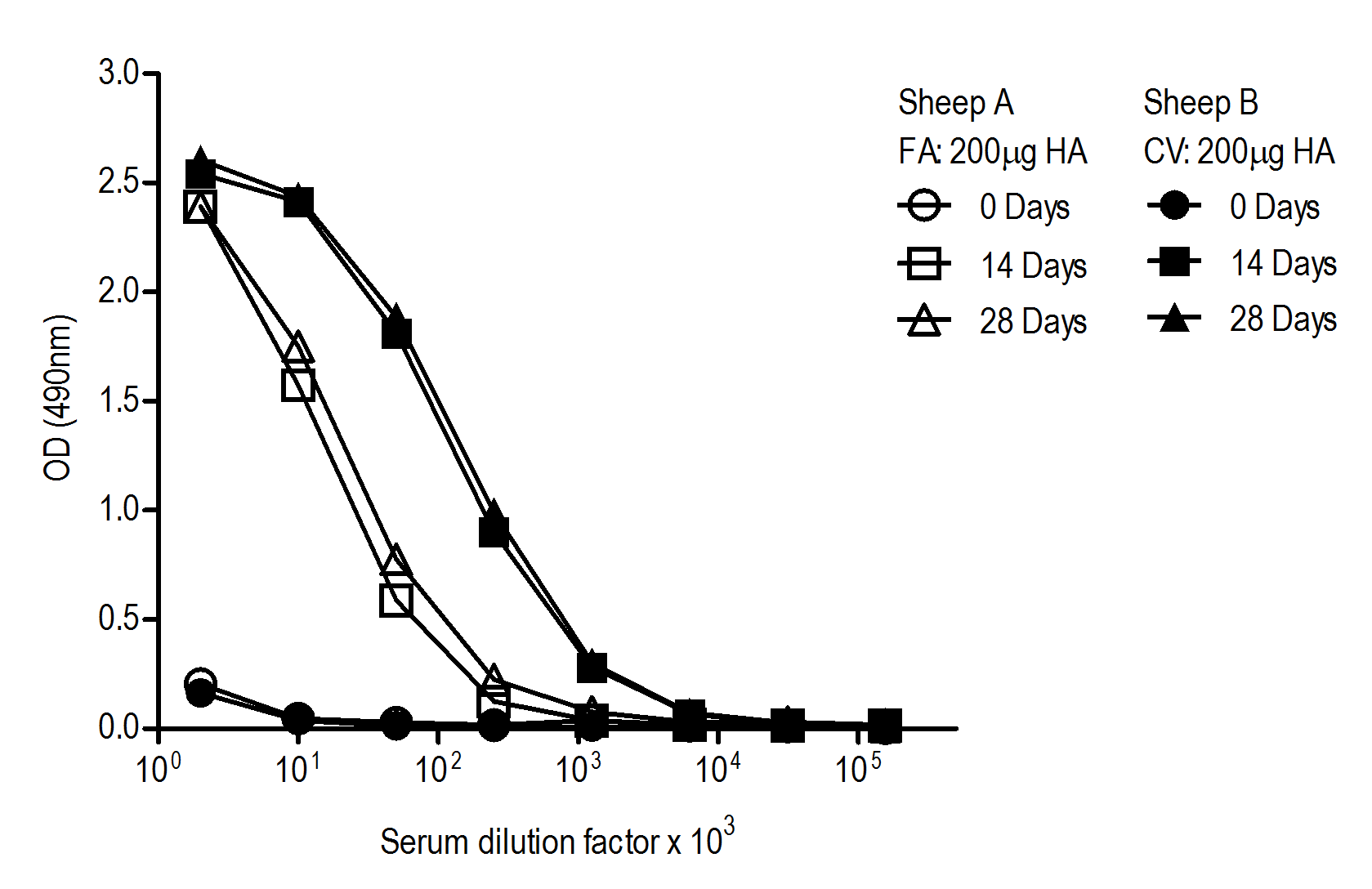

Supplement: Figure S1 — Preliminary ELISA of anti-HA ovine serum samples. An ELISA was performed on selected samples for assay development. Serial dilutions of pre-immune or hyperimmune sheep serum were added to duplicate wells of a rHA-coated ELISA plate and specific antibody was detected with HRP-linked anti-ovine IgG antibody. Signal was developed with OPD substrate until colour was visible in serum-free wells. Absorbance readings from blank wells were subtracted from all readings. The results indicated that a 1/50,000 serum dilution gave OD readings within the linear portion of the generated curve for hyperimmune samples. Consequently a 1/50,000 dilution was used in subsequent assays to assess experimental samples. (TIF) [file pone.0068895.s001.tif]
