# Supplementary material for: An Empirical Approach towards the Efficient and Optimal Production of Influenza-Neutralizing Ovine Polyclonal Antibodies Demonstrates That the Novel Adjuvant CoVaccine HT™ Is Functionally Superior to Freund's Adjuvant
Source: PLoS One. 2013 Jul 23;8(7):e68895. doi: 10.1371/journal.pone.0068895 (PMC3720891; doi:10.1371/journal.pone.0068895)
Supplement: Table S1 — Assessment of the effects of Receptor Destroying Enzyme on Haemagglutination-inhibition endpoint titres. In order to determine the effects of receptor-destroying enzyme (RDE) on endpoint HAI titres; selected serum samples were assayed with or without treatment with RDE (Sigma, 37°C, O/N). Samples were then treated with chicken red blood cells and assayed as described. Endpoint HAI titres were identical for all samples tested. (DOCX) [file pone.0068895.s002.docx]

Table S1: Comparison of Haemagglutination-Inhibition endpoints with and without the use of receptor destroying enzyme

| Sheep ID | Sample ID | HI Endpoint titre | |
| --- | --- | --- | --- |
|  |  | + RDE | - RDE |
| A – FA, 200µg HA | Day 0 | 1 | 1 |
|  | Day 14 | 3200 | 3200 |
|  | Day 42 | 3200 | 3200 |
| B – CV, 200µg HA | Day 0 | 1 | 1 |
|  | Day 14 | 3200 | 3200 |
|  | Day 42 | 12800 | 12800 |
| C – CV, 20µg HA | Day 0 | 200 | 200 |
|  | Day 14 | 800 | 800 |
|  | Day 42 | 12800 | 12800 |
